# COVID-19-Related Knowledge and Anxiety Response among Physical Education Teachers during Practical In-Person Lessons: Effects of Potential Moderators

**DOI:** 10.3390/bs12030083

**Published:** 2022-03-17

**Authors:** John Elvis Hagan, Frank Quansah, Stephen Kofi Anin, Richmond Stephen Sorkpor, Richard Samuel Kwadwo Abieraba, James Boadu Frimpong, Medina Srem-Sai, Thomas Schack

**Affiliations:** 1 Department of Health, Physical Education and Recreation, University of Cape Coast, Cape Coast PMB TF0494, Ghana; james.frimpong@stu.ucc.edu.gh; 2 Neurocognition and Action-Biomechanics-Research Group, Faculty of Psychology and Sports Science, Bielefeld University, Postfach 10 01 31, 33501 Bielefeld, Germany; thomas.schack@uni-bielefeld.de; 3 Department of Educational Foundations, University of Education, Winneba P.O. Box 25, Ghana; fquansah@uew.edu.gh; 4 Department of Industrial and Health Sciences, Faculty of Applied Sciences, Takoradi Technical University, P.O. Box 256, Takoradi WS-200-1123, Ghana; stephen.anin@ttu.edu.gh; 5 School of Public Health, Bielefeld University, P.O. Box 100131, 33501 Bielefeld, Germany; 6 Department of Health, Physical Education, Recreation and Sports, University of Education, Winneba P.O. Box 25, Ghana; rssorkpor@uew.edu.gh (R.S.S.); rskabieraba@uew.edu.gh (R.S.K.A.); mssai@uew.edu.gh (M.S.-S.)

**Keywords:** COVID-19, COVID-19-related anxiety, educational qualifications, knowledge, PE teachers, workplace safety perception

## Abstract

The COVID-19 pandemic has resulted in heightened anxiety levels among teachers, especially regarding PE teachers who are required to engage students in practical in-person or contact teaching lessons. Previous research showed that these levels of anxiety among PE teachers appeared to be explained by the interplay between COVID-19 knowledge, workplace safety perception, and educational qualification. This study assessed the relationship between COVID-19-related knowledge and anxiety response among PE teachers during such practical lessons while moderating the effects of workplace safety perception and educational qualification within the relationship. The study conveniently recruited 160 PE teachers to solicit responses through both online and printed questionnaires. Using correlation and linear regression analyses, the study revealed a significant negative relationship between COVID-19-related knowledge and anxiety response among PE teachers. The educational qualification of PE teachers did not significantly moderate the association between COVID-19-related knowledge and anxiety response. Workplace safety perception significantly moderated the association between COVID-19-related knowledge and anxiety response among PE teachers. The findings remind educational authorities about the essence of creating a positive and safe working environment conducive to academic work. Achieving this goal requires the provision of adequate COVID-19 management logistics (e.g., personal protective equipment, hand sanitizers) by educational authorities for PE teachers to maintain safety practices and optimal learning conditions.

## 1. Introduction

The impact of the COVID-19 pandemic cut across the biological, economic, health, psychological, and social dimensions of people’s lives [1]. The pandemic-associated risks of morbidity and mortality rates have become unprecedented despite the numerous counter-restrictive measures (e.g., lockdowns, physical/social distancing, quarantines), including vaccine roll-out programs [1]. Because of the unique features of COVID-19, including its virulence, large proportions of people who remain asymptomatic may still spread the virus [2]. The introduction of these measures to counter the pandemic has fundamentally changed how societies function, affecting work, family, education, and businesses [3,4]. Institutions of learning, including primary and secondary schools, colleges, and universities, with their teachers and students across societies, have been the most affected [5,6,7].

Physical education (PE) is considered one of the privileged study programs to promote individuals’ physical activity, both in the school [8,9,10] and out-of-school [11,12,13]. Unlike other study programs, PE creates an environment that fosters positive values, secures students’ engagement, and promotes positive development as well as wellbeing [14,15,16]. Due to the pandemic and the related academic interruptions, teachers were faced with an unprecedented challenge of continuing a pedagogical connection with students by quickly offering alternate instructional delivery models through new pedagogical practices such as online PE video classes, simulation classes, and virtual reality classes [17,18,19]. After over 9 months of school lockdown due to the COVID-19 pandemic, academic work began in schools [20], of which PE teachers were included and required to engage students in practical lessons in person. The resumption of schools caused widespread uncertainty, apprehension, anxiety, and fear among teachers and students within the academic environment [21]. For PE teachers and students, this psychological effect would be heightened due to the contact lessons (in-person practical classes) in which the teachers are required to engage with the students [19].

Across many developing societies, the workplace (i.e., including the teaching–learning environment for teachers) was disrupted by the COVID-19 pandemic and further complicated by scarce resources such as inadequate personal protective equipment (PPEs), teaching, and learning materials, including infrastructure, facilities, technical equipment, and other logistical aids [22,23,24,25]. Consequently, teachers are faced with enormous psychological pressure and the burden to meet instructional and other academic-related goals amidst the ongoing pandemic because of context-specific limitations [26,27]. Again, the fear of contracting the virus from the workplace setting not only threatens the teachers’ psychological wellbeing but also affects their physical, intellectual, emotional, and occupational health [28]. The emergence of the new variant, Omicron—B.1.1.529, may further compound teachers’ perceptions of safety measures amidst teaching and learning across many societies despite the implementation of numerous interventions to combat the ongoing pandemic [29]. 

Despite previous research works on the influence of COVID-19 on students’ mental health, socio-economic life, and educational pursuits in many countries [7,30,31,32,33,34], scholarly information on similar themes among teachers is sparse, especially for PE teachers who organize practical (in-person) lessons for students. Besides, the psychological reactions that may be elicited among teachers may also be country- and/or region-specific. With teachers expected to have considerable content and pedagogical knowledge in their respective domains, research linking their COVID-19-related knowledge and anxiety-related responses using educational qualification and workplace safety perception as moderators toward the maintenance of their overall psychological health during this unprecedented period is essential. 

Previous studies have already proven the connection between COVID-19-related knowledge and the associated anxiety response among frontline healthcare workers, college students, adults with chronic conditions, and residents of mainland China over 18 years old. However, the existing findings are inconsistent. While some studies identified negative associations [35,36,37], others established positive linkages [38] as well as no association [39]. These discrepancies might be due to other confounding variables such as workplace safety perception, educational qualifications, and other methodological limitations that may alter identified associations [40,41,42,43,44,45]. Recently, Quansah et al. [46] assessed the perceived safety in the PE learning environment and associated anxiety factors during the ongoing pandemic among university students. Findings showed that PE students perceived the practical (in-person or contact teaching) lesson environment as unsafe and reported moderate to high levels of anxiety during these practical lessons. Quansah and coworkers emphasized that the practical PE lesson environment could serve as a potential gateway for COVID-19 acquisition and transmission with heightened anxiety levels. Whether these same experiences would manifest among PE teachers amidst the ongoing COVID-19 pandemic is yet to be documented. Regarding educational qualification, quite a number of scholars [42,47] underscored that educational qualification is positively associated with COVID-19-related knowledge. These authors further suggested a possible interaction between educational qualification and knowledge. Hence, the educational qualification has a probability of moderating the relationship between knowledge and anxiety amidst COVID-19. 

To the best of the authors’ knowledge, no study was previously conducted on these moderation effects and has concurrently measured these research variables in one single design. The findings of the study may unearth useful information concerning teachers’ knowledge gaps on COVID-19 and their psychological reactions that may require urgent attention to help manage the ongoing pandemic. The study could also guide the implementation of appropriate interventions aimed at safeguarding teachers’ mental health and overall wellbeing. The rationale for the current study is to: (1) examine the relationship between COVID-19-related knowledge and anxiety response among PE teachers during practical lessons; (2) assess the moderating role of educational qualification of PE teachers in the relationship between COVID-19-related knowledge and anxiety response; and (3) evaluate the moderating role of workplace safety perception in the relationship between COVID-19-related knowledge and the anxiety response of PE teachers.

## 2. Materials and Methods

### 2.1. Research Design and Approach

An analytical cross-sectional survey design of the quantitative research approach was used to examine the relationship between COVID-19-related knowledge and anxiety response among PE teachers during practical (in-person) lessons.

### 2.2. Participants’ Selection

The study targeted all PE teachers (*n* = 178) from the senior high schools (SHS) in the Central Region of Ghana. PE teachers who were readily available, accessible, ready, and willing to be part of the study were recruited from the various schools in the region. One hundred and sixty (*n* = 160), representing a response rate of 89.9% of participants comprising teachers with certificate ‘A’—9, Diploma—38, Bachelor—82, and Master’s—31 were conveniently selected for the study. None of the participants had a PhD qualification. Additionally, the selected teachers had varied years of working experience: 19 teachers had less than a year working experience, 30 reported 1–2 years working experience, 37 indicated 3–4 years of working experience, and those who have taught above 5 years numbering up to 74 (see Table 1). Out of 160 teachers, 106 of them were males, and 54 were females. 

### 2.3. Instrumentation

The questionnaire was developed in English with four sections. Part one of the instrument was made up of demographic variables such as gender, level of education, and years of working experience. The second part of the instrument had six items that measured teachers’ knowledge on COVID-19. The third part also had six items related to COVID-19-related anxiety. The final part had five items on workmanship climate.

### 2.4. Study Variable Measurements

#### 2.4.1. Knowledge of COVID-19

COVID-19-related knowledge in this study was measured using six items developed and validated by the investigators. The items covered issues of COVID-19 infections, mode of transmission, symptoms, and management of the condition. For example, the participants were asked to identify (from already provided options) the major ways in which people were becoming infected with COVID-19. A similar question was posed to the participants to also identify (from already provided options) the major symptoms exhibited by COVID-19-infected persons. Other items which demanded a simple True or False response include: “COVID-19 can spread through persons via asymptomatic carriers”, “Measures such as early detection, isolation, contact tracing, social distancing, and wearing of face masks helps keep the infections and transmission of COVID-19 at a minimum”, “Though COVID-19 has no absolute treatment, the symptoms can be managed very well”, “Keeping away from crowded areas helps to prevent and reduce the rate of COVID-19 infections and transmissions”. The items measuring knowledge were factual, and responses were scored as right (i.e., a score of 1 assigned) or wrong (i.e., a score of 0 assigned). The right answers to the items measuring COVID-19 knowledge were supported by previous studies [48,49,50,51]. The items were scored out of a total score of six, with large values indicating a high COVID-19 knowledge level and lower values depicting low knowledge on COVID-19.

The items were calibrated to ensure that they could produce valid and reliable responses. After the items were carefully developed and scrutinized by other experts, pilot testing was conducted using 31 teachers who were conveniently sampled through online means. A test re-test reliability procedure was carried out to assess the stability of the trait and to be sure that participants do not guess on the items [52]. A test re-test reliability yielded a reliability estimate of 0.823, indicating a sufficient level of consistency [53]. After the main data collection, the internal consistency of the items using the Kuder–Richardson 21 reliability estimate was conducted [54], and this yielded a reliability estimate of 0.739, which was also deemed adequate [53].

#### 2.4.2. COVID-19-Related Anxiety

COVID-19-related anxiety was conceptualized as the degree to which the participants experienced a number of listed symptoms during practical lessons amid the COVID-19 pandemic. The anxiety scale comprised six items which were adapted from Beck et al.’s [55] anxiety scale, which has widespread use. Since the participants in this study were not hospitalized or did not have any condition, the researchers only sampled items that reflected non-clinical indicators. This approach to item generation and adaptation has been used in recent times by other researchers like Quansah et al. [46]. The statements include: “I feel nervous”, “I feel unsteady”, “I have self-doubts”, “I feel very much concerned”, “I feel unrelaxed”, and “I fear the worst happening”. The items had response options ranging from 0–3, with 0 indicating ‘not at all’, 1 depicting-somewhat, 2 representing moderately, and 3 indicating very much so. The psychometric properties of the anxiety scale were reported to be adequate, with an omega reliability estimate greater than 0.71 [46]. High scores on the anxiety scales depict a high level of anxiety among the participants during practical lessons amid COVID-19 and vice versa.

#### 2.4.3. Workplace Safety Perception 

Workplace safety perception was conceptualized as the degree to which the teaching environment (i.e., practical lesson class in the context of this research) is perceived as safe from COVID-19 infection and transmission. A set of five items was developed by the investigators to measure this construct. The items comprised: “Does the institution provide the necessary protective equipment to prevent the infection and transmission of COVID-19 during practical lessons?”, “Are you comfortable engaging students in practical lessons amidst this COVID-19 outbreak?”, and “Do you feel safe teaching in practical lessons amid COVID-19 pandemic”. The participants were required to respond to the items using a “Yes” or “No”. For purposes of analysis, “yes” and “no” responses were given a weight of 1 and 0, respectively. The measurement of perceived workplace safety and its associated scoring was deeply rooted in the framework of optimal/maximum testing [56,57]. As such, scoring for this variable was factual; whether the workplace environment was safe or unsafe. With this, the measurement literature [58,59] supports and justifies the use of 0 vs. 1 to depict the absence vs. presence of the trait being measured. The scale was scored out of five, with large scores representing the positive perception of workplace safety and low scores depicting the negative perception of workplace safety. Specifically, a score greater than or equal to four was classified as a positive perception of workplace safety, whereas a score below four was assigned a negative perception of workplace safety. A reliability estimate of 0.72 was obtained using the Kuder–Richardson 21 reliability test approach, which was deemed sufficient [52].

#### 2.4.4. Educational Qualification

Educational qualification was measured by the participants (i.e., teachers), indicating the highest qualification they use for their teaching profession. A single item was composed with the question: “What is the highest level of education you have completed?”. The options which were provided were: Certificate, Diploma, Bachelor’s degree, Master’s degree, and Doctor of Philosophy (PhD) or doctorate degree. This variable was categorical; therefore, it was treated as such for analysis purposes. The measurement approach to educational qualification was adopted from the United Nations Educational, Scientific, and Cultural Organization [UNESCO] [60] definition of educational qualification in the International Standard Classification of Education (ISCED 2011) and International Standard Classification of Education: Fields of Education and Training 2013 (ISCED-F 2013).

### 2.5. Data Collection Procedure

Ethical standards were applied in the conduct of the study. Ethical clearance was provided by the Institutional Review Board, University of Cape Coast, Ghana, with a reference number of UCCIRB/EXT/2020/25. Permission was also sought from the heads of the selected institutions, who acted as gatekeepers, to gain access to the research site [61]. An earlier contact was made with the headteachers through the regional PE coordinators to establish rapport and as well introduce to the PE teachers the general aim of the study. The benefit of the research and its impact on other relevant stakeholders were discussed with the potential participants. Contact details, including the telephone numbers and email addresses of the participants, were documented. After creating a rapport with the PE teachers in the various SHS, arrangements were made with the participants regarding when they would be available to respond to the instrument. While some participants agreed that the administration should be carried out in paper-and-pencil form, others insisted that they wished to respond to the instrument online, leading to the creation of online versions of the questionnaire. Those who participated in the online survey were given 24 h to respond, whereas those who responded to the printed instrument were required to respond to the instrument at a place convenient to them and hand it over to the investigators.

In whichever form the administration was carried out, consent forms were signed by each participant before they responded to the instrument. Other ethical considerations such as confidentiality, anonymity, volition, protection from psychological or emotional harm, and privacy were adhered to. For example, participants who decided to quit the survey were allowed to do so, and consequently, six participants opted out of the study when they were sent the online version of the instrument. Similarly, two participants who were administered the printed instruments only responded to the demographic information. These instances led to the obtaining of 160 out of 168 cases. The data were collected during the period when schools had resumed teaching and learning activities after the closure of schools for some months. The entire data collection lasted for two months.

### 2.6. Statistical Analyses

The analyses of the data were structured in different phases based on the objectives of the study. The descriptive analyses were first carried out to explore the variables and understand the nature of the variables as used within the context of the research. The mean and standard deviation for the continuous variables were explored, followed by the relationship existing between the variables (e.g., biserial correlation and Pearson product–moment correlation). The first objective, which sought to examine the relationship between COVID-19-related knowledge and anxiety, was addressed by performing a simple linear regression analysis. For objectives two (i.e., examine the moderating role of educational qualification in the relationship between COVID-19 knowledge and anxiety response during practical lessons) and three (i.e., assess the moderating role of workplace safety perception in the relationship between COVID-19-related knowledge and anxiety response of PE teachers), moderation analysis using PROCESS software by HAYES was performed. For all the inferential analyses, the bootstrapping approach with 5000 bootstrap samples was used for the parameter estimation. 

## 3. Results

### 3.1. Descriptive Analysis

The descriptive statistics for the variables under investigation were explored to understand the nature of the variables. The means and standard deviations, as well as the correlational indices of each pair of the variables, were reported. The details are shown in Table 2.

Regarding the mean and standard deviation estimates, workplace safety perception had a mean of 2.50 with a standard deviation of 1.49, signaling a negative perception of workplace safety during the COVID-19 era (see Table 2). For anxiety level, the participants reported a moderate COVID-19-related anxiety level (*M* = 1.71, *SD* = 0.54). The knowledge level of the participants was quite high, with a mean of 4.74 and a standard deviation of 1.01. Regarding the correlational outcome, the data showed a significant negative relationship between workplace safety perception and COVID-19-related anxiety (*r* = −0.199). COVID-19-related anxiety and knowledge of PE teachers were significantly and negatively related (*r* = −0.251). The analysis also showed a positive significant link between educational qualification and COVID-19-related knowledge (*r* = 0.137). No significant relationship was found between COVID-19 knowledge and workplace safety perception. Similarly, no significant association was found between educational qualification and workplace safety perception. 

### 3.2. Relationship between COVID-19-Related Knowledge and Anxiety Response among PE Teachers during Practical Lessons

This study first sought to establish the relation between COVID-19-related knowledge and anxiety response among PE teachers. The details of the simple linear regression analysis are shown in Table 3 and Table 4.

The results in Table 3 highlight the model summary and fit statistics of the regression results. It was revealed from the analysis that the data, which comprised COVID-19 knowledge and COVID-19-related anxiety fit the model, *F*(1, 158) = 10.653, *p* = 0.001. The outcome of the analysis also showed that COVID-19 knowledge explained about 6.3% of the variability in COVID-19-related anxiety.

The results revealed that knowledge negatively predicted COVID-19-related anxiety among PE teachers during practical lessons, *B* = −0.134, *SE* = 0.041, *t* = −3.264, Boot*CI* (−0.216, −0.053) (see Table 4). This outcome suggests that PE teachers with a high level of COVID-19 knowledge have higher chances of experiencing low COVID-19-related anxiety. PE teachers with low knowledge of COVID-19 are more likely to exhibit high levels of anxiety during practical lessons.

### 3.3. The Moderating Role of Educational Qualification in the Relationship between COVID-19 Knowledge and Anxiety Response during Practical Lessons

This research also sought to moderate the educational qualification of PE teachers in the relation between knowledge on COVID-19 and anxiety response during practical lessons. Table 5 present the details of the moderation analysis outcome.

The outcome of the analysis in Table 5 showed that none of the levels of the moderator variable (educational qualification) significantly moderated the relationship between knowledge on COVID-19 and anxiety response of PE teachers; Diploma (W1) * knowledge [*B* = 0.142, *SE* = 0.230, Boot*CI* (−0.312, 0.596), Bachelor’s degree (W2) * knowledge [*B* = −0.002, *SE* = 0.219, Boot*CI* (−0.434, 0.431), Master’s (W3) * knowledge [*B* = 0.000, *SE* = 0.231, Boot*CI* (−0.457, 0.457). The results generally found that educational qualification failed to moderate the relationship between knowledge on COVID-19 and the anxiety response of PE teachers.

### 3.4. The Moderating Role of Workplace Safety Perception in the Relationship between COVID-19-Related Knowledge and Anxiety Response of PE Teachers

The last objective of this research sought to examine the moderating role of workplace safety perception in the link between COVID-19 knowledge and anxiety response of PE teachers. Table 6 outline the details of the analysis.

**Figure 1:**
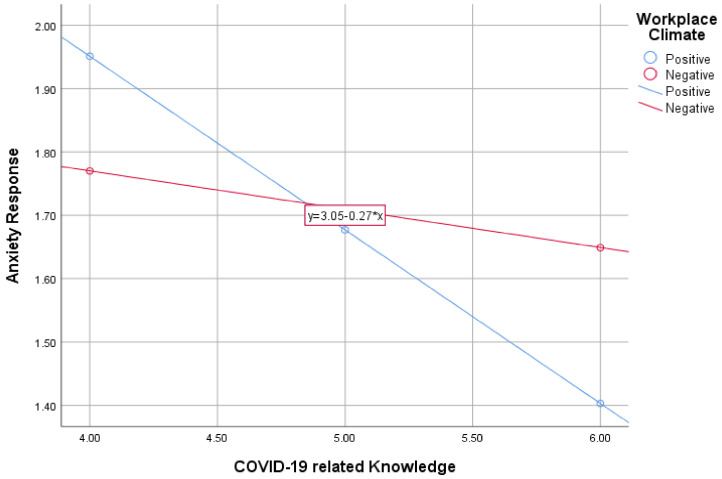
Probing graph on the moderating role of workplace safety perception in the link between COVID-19 knowledge and anxiety.

The outcome of the moderation analysis revealed that workplace safety perception significantly moderated the relationship between COVID-19 knowledge and anxiety response, *B* = 0.214, *t* = 2.434, Boot*CI* (0.040, 0.387). (see Table 6) The significant moderation analysis was further probed using the graph in Figure 1. 

The findings from the graph showed that a positive perception of workplace safety, with a much steeper regression line, strengthens the negative relationship between COVID-19-related knowledge and the anxiety response of PE teachers during practical lessons. As can be observed, enhancing PE teachers’ knowledge on COVID-19 with a positive perception of workplace safety results in a significantly decreasing level of COVID-19-related anxiety during practical lessons. On the contrary, increasing the level of COVID-19 knowledge of PE teachers in a negative workplace does not meaningfully reduce the anxiety level of these PE teachers.

## 4. Discussion

This study sought to examine the relationship between COVID-19-related knowledge and anxiety response among PE teachers during practical in-person or contact teaching lessons, and as well assess the independent moderating roles of the perceived safety of work environment and educational qualification of PE teachers in the relationship between COVID-19-related knowledge and anxiety response. The study findings showed that there was a significantly negative relationship between COVID-19-related knowledge and anxiety response among PE teachers. Educational qualification of PE teachers did not significantly moderate the association between COVID-19-related knowledge and anxiety response. Workplace safety perception significantly moderated the association between COVID-19-related knowledge and anxiety response among PE teachers. 

PE teachers with a high level of knowledge exhibited lower levels of COVID-19-related anxiety as similarly observed in some studies conducted amongst midwifery students in Turkey [62], COVID-19 patients in Turkey [63], college students in China [64], and the general public and health workers in Qatar and the Middle East [65]. This negative association observed between COVID-19 knowledge levels and anxiety as a psychological health outcome was alluded to various population and/or situation-specific reasons. Notwithstanding, it is plausibly logical to agree with the postulate that the more knowledgeable one is about an impending and/or imaginary danger or threat, the less apprehensive or anxious one is likely to be [66,67,68]. For instance, in a study of couples undergoing fertility treatment who had high levels of correct COVID-19-related knowledge (mode of spread, common symptoms, and preventive measures), they exhibited lower levels of anxiety response, especially because they believed that compliance with the preventive measures was more likely to secure their health while continuing to pursue their goal of childbirth [69]. Therefore, it is suggestive that factors that facilitate COVID-19 knowledge level are more likely to inversely influence anxiety response [36,38,51,68]. 

Several factors such as the source of information about COVID-19, gender, and intolerance of uncertainty, among others, are shown to significantly moderate the relationship between COVID-19-related knowledge and anxiety [64,70,71,72]. A negative relationship between COVID-19-related level of knowledge and anxiety response was observed irrespective of the level of education of the PE teachers in this study. This non-significant moderating effect of educational status on the relationship between COVID-19-related knowledge levels and anxiety was similarly observed in other studies [39,43,45]. Some explanations for this non-significant moderating effect observed suggest that irrespective of an individual’s level of knowledge, the COVID-19 pandemic may continually generate fear and anxiety among people after its emergence and associated mortality metrics [66]. Lin et al. [39] also reported similarities in the levels of anxiety amongst the lay population in China comparable to the different categories of health workers (doctors, hospital administrative staff, and allied health workers) in Hong Kong [73]. Anxiety is a natural psychological response to real and/or imaginary threats [74]. Therefore, it is conceivable that irrespective of one’s level of education or job description and risk levels, educational status does not seem to have an intervening or mitigating role in the expression of this psychological state or trait. It is, therefore, not surprising that the level of anxiety response expressed by PE teachers during practical lessons was not influenced by their educational status. However, higher levels of educational attainment were reported to have a protective effect against anxiety and depression cumulatively throughout life [75]. Indeed, one’s education level may prove helpful in the deployment of psychological interventions (e.g., positive self-talk, mental rehearsal, cognitive restructuring, and counselling) for PE teachers and, therefore, facilitative for optimum instructional delivery services to students [46]. Couples undergoing fertility treatment who had higher levels of correct COVID-19-related knowledge correspondingly exhibited lower levels of anxiety response, probably also because their goal of seeking to have children provided additional counteraction against the fear of COVID-19. Interestingly those who had higher educational qualifications had higher odds of continuing with their fertility treatments amid the pandemic compared to those with lower educational attainment [69].

A positive workplace safety perception and/or PE learning environment during practical or in-person sessions possibly serves as a shield against the effect of a low level of COVID-19-related knowledge on anxiety levels through its mediating role [51,76]. PE teachers with low knowledge levels of COVID-19 working within a positive working environment during practical or in-person lessons in this study showed low anxiety levels, as similarly reported in other studies [46,77,78]. One possible reason for the significant moderating effects of perceived workplace safety is that the perception that one has about the risk of contracting COVID-19 and other potentially debilitative psychological health outcomes at the workplace compared to being at home or away from in-person engagements induces or triggers various negative emotional responses including anxiety, fear, panic, and worry [79].

Studies that reported positive linear associations between COVID-19-related knowledge levels and anxiety intimated that persons who are more aware of the uncertainties surrounding COVID-19 are more likely to experience high levels of anxiety [38,51,80]. Due to the COVID-19 pandemic-related unparalleled fear, apprehension, and nervousness, having more knowledge on the seriousness of the consequences of the virus could have caused the heightened anxiety levels. This contradictory or counterintuitive finding may be due to the moderating effects of some other factors unaccounted for in the analysis, choice of measurement scales for the independent and dependent variables of interest, and some possible sources of biases (confounders, reporting, covariate selection) arising from methodological flaws and/or disparities compared with this study.

### 4.1. Strengths and Limitations

This study appears to be the first attempt to examine the moderating roles of epidemiologically significant factors such as educational qualification and workplace safety perception on the putative association between the level of COVID-19-related knowledge and anxiety amongst PE teachers during practical lessons. The cross-sectional design used in this study precludes the possibility of any causal inference. The generalization of the findings of this study should be alluded to with caution, given that the study participants are not representative of all PE teachers because they were conveniently sampled from SHS in the Central region and not the whole county, including other study fields. However, the insights gained from these participants offer an opportunity for future studies with enhanced representativeness. Errors introduced by over and/or under-reporting biases, confounders, endogeneity biases inherent in cross-sectional study designs may not be entirely ruled out. Notwithstanding these limitations, this study contributes significantly to the dearth of literature on the relationship between COVID-19 knowledge level within academic or pedagogical settings in Ghana and anxiety as a psychological health outcome amongst teachers during their instructional delivery. Future research should be conducted to examine the impact of COVID-19 knowledge level and workplace safety perception on other psychological health outcomes [79] with more robust study designs such as longitudinal analyses and multi-center studies, as espoused by O’Connor et al. [81].

### 4.2. Practical Implications 

The pandemic related threats to learning and the overall wellbeing of teachers, including students and other analogous staff, should remind educators and policymakers about the essence of creating a positive and safe working environment conducive for academic work. This study sheds light on the importance of creating a safe working environment or climate and adequate COVID-19 knowledge empowerment as key interventions against possibly debilitating psychological health outcomes such as anxiety amongst PE teachers and other persons who are actively involved in instructional delivery and pedagogical practices in educational institutions and other learning centres. Although COVID-19 has facilitated some unpleasant experiences, this pandemic period offers opportunities for transformational changes. More flexible and responsive systems are required for teaching and learning. The integration of virtual and digital learning for in-person school environments and the collaborative use of the innovative hybrid modes of curriculum delivery may be useful for achieving academic-related goals and boosting workplace safety, including overall wellbeing. Achieving this goal also requires the provision of adequate COVID-19 management logistics (e.g., PPEs, hand sanitizers) by educational authorities for PE teachers to maintain safety practices and learning conditions.

## 5. Conclusions

The outcome of this research stresses the pivotal role of workplace safety perception and knowledge on COVID-19 in explaining the variability in anxiety levels of PE teachers during practical lessons amidst the COVID-19 pandemic. Whereas PE teachers with high knowledge levels on COVID-19 exhibited lower levels of COVID-19-related anxiety, this relationship becomes more strengthened in a workplace safe from COVID-19 infections and transmissions. It is of essence to emphasize that both enhanced knowledge on COVID-19 and a safe working environment are critical factors in reducing anxiety among PE teachers during practical lessons. The absence of one of these conditions (i.e., enhanced knowledge on COVID-19 and positive perception of workplace safety) intensifies the anxiety incidence among PE teachers during practical lessons, especially in these COVID-19 times. The increasing science-driven advertisement on television, radio, social media platforms, churches, mosques, hospitals, and community centres, is an attempt by all stakeholders to improve populace knowledge on COVID-19 in terms of the nature of the virus. However, this only perhaps helps individuals to prevent infection and transmission of the virus but does not necessarily help their psychology and further creates psychological distress such as extreme anxiety, as shown in this study. Thus, the need for creating a positive and safe work environment is paramount. This study calls on relevant stakeholders, such as the government, World Health Organization (WHO), Non-governmental Organizations (NGO), and administrators of educational institutions, not to only invest in creating awareness and improving the COVID-19-related knowledge of all persons, but to also liaise with the management of educational institutions and other employees to create a safe working environment for PE teachers and other teaching staff. 

## Figures and Tables

**Table 1:** Sociodemographic data of PE teachers in terms of gender, level of education, and years of teaching.

Gender	No. (%)	Level of Educ.	No. (%)	Years of PE Teaching	No. (%)
Male	106 (66.25)	Certificate	9 (5.6)	Less than 1 year	19 (11.9)
Female	54 (33.75)	Diploma	38 (23.8)	1–2 years	30 (18.8)
		Bachelor	82 (51.2)	3–4 years	37 (23.1)
		Master’s	31 (19.4)	Above 5 years	74 (46.3)
Total	160 (100)	--	160 (100)	--	160 (100)

**Table 2:** Correlations, means, and standard deviations for the variables in this study.

Variables	Workplace Safety Perception	Anxiety	Knowledge	Educational Qualification
Workplace safety perception	1			
Anxiety	−0.199 *	1		
Knowledge	0.094	−0.251 **	1	
Educational qualification	−0.051	0.128	0.137 *	1
Mean	2.50	1.71	4.74	--
Standard Deviation	1.49	0.54	1.01	--

* Correlation is significant at the 0.05 level (2-tailed). ** Correlation is significant at the 0.01 level (2-tailed).

**Table 3:** Model summary and fit statistics of the relationship between COVID-19-related knowledge and anxiety.

Model		Sum of Squares	df	Mean Square	F	Sig.	R^2^
1	Regression	2.933	1	2.933	10.653	0.001	0.063
	Residual	43.496	158	0.275			
	Total	46.429	159				

Criterion Variable: COVID-19-related anxiety; Predictors: (Constant), knowledge.

**Table 4:** Coefficient of the prediction of COVID-19 knowledge on COVID-19-related anxiety.

Model		*B*	*SE*	Beta	*t*	*p*	LLCI	ULCI
1	(Constant)	2.349	0.200		11.767	0.000	1.955	2.743
	Knowledge	−0.134	0.041	−0.251	−3.264	0.001	−0.216	−0.053

*B*—Unstandardized Coefficient; *SE*—Standard Error; LLCI—Lower Limit Confidence Interval; ULCI—Upper Limit Confidence Interval.

**Table 5:** Moderation effect of educational qualification in the relationship between COVID-19 knowledge and anxiety.

	*B*	*SE*	*t*	*p*	BootLLCI	BootULCI
Constant	2.556	0.930	2.747	0.007	0.717	4.394
Knowledge	−0.167	0.211	−0.790	0.431	−0.584	0.250
W1	−0.825	1.014	−0.814	0.417	−2.827	1.178
W2	−0.058	0.979	−0.059	0.953	−1.992	1.876
W3	0.158	1.030	0.153	0.878	−1.877	2.192
W1 * Knowledge	0.142	0.230	0.616	0.539	−0.312	0.596
W2 * Knowledge	−0.002	0.219	−0.007	0.995	−0.434	0.431
W3 * Knowledge	0.000	0.231	0.002	0.999	−0.457	0.457

Model Summary: *R*^2^ = 0.125; *F*(7, 152) = 3.116, *p* = 0.004. W1—Diploma; W2-Bachelor’s degree; W3—Master’s degree. *B*—Unstandardized Coefficient; LLCI—Lower Limit Confidence Interval; ULCI—Upper Limit Confidence Interval; *SE*—Standard Error; Asterisk (*) represent interaction sign.

**Table 6:** Moderation effect of workplace safety perception in the relation between COVID-19 knowledge and anxiety.

	*B*	*SE*	*t*	*p*	BootLLCI	BootULCI
Constant	3.048	0.369	8.266	0.000	2.319	3.776
Knowledge	−0.274	0.071	−3.844	0.000	−0.415	−0.133
W1	−1.035	0.440	−2.351	0.020	−1.905	−0.165
W1 * Knowledge	0.214	0.088	2.434	0.016	0.040	0.387

W1—Negative perception of workplace safety; Model Summary: *R*^2^ = 0.098; *F*(3, 156) = 5.622, *p* = 0.001. *B*—Unstandardized Coefficient; *SE*—Standard Error; LLCI—Lower Limit Confidence Interval; ULCI—Upper Limit Confidence Interval; Asterisk (*) represent interaction sign.

## Data Availability

The data are available upon reasonable request through the corresponding author.
